# Reclassification of VUS Using ACMG/AMP Criteria Adapted for Sarcomeric Genes Related to Hypertrophic Cardiomyopathy: Resolution Rate and Considerations

**DOI:** 10.1155/humu/6500093

**Published:** 2025-11-29

**Authors:** Silvia Caroselli, Giulia Corona, Marco Fabiani, Martina Manzoni, Caterina Micolonghi, Camilla Savio, Aldo Germani, Stefania Bragliola, Valeria Maselli, Speranza Rubattu, Beatrice Musumeci, Giacomo Tini, Vincenzo Visco, Simona Petrucci, Valeria Novelli, Maria Piane

**Affiliations:** ^1^Department of Experimental Medicine, Sapienza University of Rome, Rome, Italy; ^2^Unità di Genetica Cardiovascolare, Centro Cardiologico Monzino IRCCS, Milan, Italy; ^3^UOD Genetica Medica, Sant'Andrea University Hospital, Rome, Italy; ^4^Department of Clinical and Molecular Medicine, Sapienza University of Rome, Rome, Italy; ^5^UOC Cardiologia, Sant'Andrea University Hospital, Rome, Italy; ^6^Angio-Cardio-Neurology Division, IRCCS Neuromed, Pozzilli, Italy

## Abstract

**Background:**

Genetic testing is valuable to confirm molecular diagnosis in nearly 60% of cases suspected of hypertrophic cardiomyopathy (HCM). However, the interpretation of variants, especially those of uncertain significance (VUSs), remains challenging for laboratories and clinicians. In April 2024, the ClinGen Cardiomyopathy Variant Curation Expert Panel (VCEP) adapted the ACMG/AMP criteria for eight of the sarcomeric genes (*MYH7*, *MYBPC3*, *TNNI3*, *TNNT2*, *TPM1*, *ACTC1*, *MYL2*, and *MYL3*), providing a refined framework for variant interpretation in these genes. This retrospective study re-evaluated 69 VUSs identified in 84 HCM patients between 2017 and 2024, aiming to resolve uncertainty and reduce the VUS rate.

**Methods:**

Here, two groups of curators reinterpreted variants with the most recent data using the Cardiomyopathy VCEP specifications until a consensus was reached. To streamline the process, we created a semiautomated decision support tool based on these gene-specific rules.

**Results:**

The application of the Cardiomyopathy VCEP specifications resulted in the reclassification of 17.4% (*N* = 12/69, 95% CI: 10.2%–28.0%) of VUS, whereas the new data alone were not sufficient. Out of the reclassified variants, 91.7% (*N* = 11/12) were downgraded to benignity (involving 17 patients), and 8.3% were upgraded to pathogenicity (involving one patient), with a mean reclassification time of 68.3 months, corresponding to 5.7 years. The most applied criteria were related to population (PM2 = 55%; BA1/BS1 = 16%), bioinformatic prediction (PP3 = 45%; BP4 = 25%), and critical domains (PM1 = 21%). However, most codes suffer from a lack of evidence (segregation data, functional assays, and case-control studies). When comparing this curation with classifications in public databases, 13.3% (*N* = 8/60) and 16.2% (*N* = 11/68) of variants listed as having inconclusive significance in ClinVar and CardioClassifier were, respectively, reclassified in this study.

**Conclusion:**

Using gene-specific ACMG/AMP criteria reduces the rate of VUS, increasing diagnostic yield, and informing clinical management in the context of HCM. Nonetheless, ongoing efforts to generate evidence and promote standardization remain essential to improve variant interpretation.

## 1. Introduction

Hypertrophic cardiomyopathy (HCM) is a genetic myocardial disorder defined as the presence of increased left ventricular wall thickness or mass in the absence of abnormal loading conditions [1]. In familial forms of HCM, inheritance is primarily autosomal dominant, although it is often characterized by reduced penetrance and wide variability in age of onset and disease severity [2].

Genetic testing has emerged as a pivotal tool in both the diagnosis and management of HCM patients. As highlighted in the 2023 ESC guidelines [1], the identification of a damaging variant can guide cardiologists in clinical decision-making, encompassing family screening, risk stratification for sudden cardiac death, implementation of targeted therapeutic interventions, and reproductive advice and management [2–4].

The ClinGen Gene Curation Expert Panel (GCEP) has determined which genes are associated with the HCM phenotype and should therefore be included in HCM testing. The number of genes with definitive evidence for HCM has recently been updated to 16, encompassing sarcomeric and nonsarcomeric genes [5, 6]. Of these, the first eight sarcomeric genes showing definitive relationships were *MYH7*, *MYBPC3*, *TNNI3*, *TNNT2*, *TPM1*, *ACTC1*, *MYL2*, and *MYL3*, while only recently, *TNNC1* has been upgraded to a ninth sarcomeric gene definitively associated with HCM. The eight core genes encode cardiac-specific isoforms of sarcomeric proteins, which are considered the fundamental units of myocardial contractility. Alterations of any of the sarcomeric proteins due to deleterious variants trigger a series of molecular (e.g., altered transcriptomics and metabolomics) and histological (e.g., hypertrophy and fibrosis) changes, leading to the clinical manifestations of HCM (e.g., arrhythmia and heart failure) [7].

However, assessing the pathogenicity of a specific alteration is a challenging process involving the collection and evaluation of multiple data from different sources. In 2015, to address this issue, the American College of Medical Genetics and Genomics (ACMG) and the Association for Molecular Pathology (AMP) published guidelines [8] to harmonize this process, primarily for genes that cause Mendelian disorders. In brief, this standardized framework is articulated in 28 codified criteria, ranging from population frequency to functional evidence [9], and their combined application results in a five-tier system [10] to define the clinical significance of a variant. Pathogenic and likely pathogenic (P/LP) variants are considered deleterious and disease-causing and thus correspond to a positive result in diagnostic testing. Benign and likely benign (B/LB) variants are considered neutral and unlikely to cause disease and thus correspond to negative results. Variants of uncertain significance (VUSs) indicate insufficient or conflicting evidence to determine their effect. This is considered an inconclusive result, defined as “not actionable” for clinical purposes, meaning it should not be used for clinical management or cascade testing. However, it often creates ambiguity for both healthcare professionals and patients. It is estimated that approximately 10%–20% of patients undergoing genetic screening for HCM carry at least one VUS [11], and current high-throughput genetic testing methods (e.g., gene panels and exome sequencing based on next-generation sequencing [NGS]) are identifying an increasing number of them.

To improve the process of variant interpretation, the ClinGen consortium organizes Variant Curation Expert Panels (VCEPs) to assess scientific evidence and provide expert-based recommendations to improve the consistency and accuracy of genetic diagnostics. In 2018, the Cardiomyopathy VCEP was recruited to adapt the ACMG/AMP classification criteria to HCM-associated genes. Initially focused on *MYH7* [12], specifications for variant interpretation in core sarcomeric genes were released in April 2024, including a new release of *MYH7* (available at [13]).

These adaptations address whether, how, and to what degree of strength the ACMG/AMP criteria should be applied in the context of HCM. Briefly, four criteria do not report any changes, remaining as general recommendations (PS1, PM4, PM5, and BP7), while eight criteria are no longer applicable (PM3, PP4, PP5, BS2, BP1, BP3, BP5, and BP6). Furthermore, 13 criteria become disease-specific (PS2, PS3, PS4, PM2, PM6, PP1, PP3, BA1, BS1, BS3, BS4, BP2, and BP4), and three criteria become gene-specific (PVS1, PM1, and PP2) by providing a detailed description or a reference threshold for applicability. In detail, the gene/disease-specific adaptations include relevant codons in each gene (PM1) and the thresholds for defining low and high frequency in the general population (PM2, BA1, and BS1), as well as for predicting deleterious and benign effects (PP3/BP4). Moreover, an increase in the degree of strength is now required as the evidence for functional assays (PS3/BS3), for case-control studies (PS4), and for variant cosegregation (PP1) increases, rather than a unique degree only. For the sake of completeness, the applicability rules defined by Cardiomyopathy VCEP are reported in Table 1.

Following the release of updated recommendations, we undertook a systematic re-evaluation of variants previously classified as VUSs, in accordance with established guidelines [17]. Additionally, we provided the first critical evaluation of the application of the ACMG/AMP criteria adapted for HCM-related sarcomeric genes.

## 2. Methods

### 2.1. Study Design and Participants

We retrospectively examined 69 genetic variants in core sarcomeric genes (*MYH7*, *MYBPC3*, *TNNI3*, *TNNT2*, *TPM1*, *ACTC1*, *MYL2*, and *MYL3*), referred between January 2017 and March 2024 as VUS by two Cardiovascular Genetic Units from AOU Sant'Andrea in Rome (SA) and Centro Cardiologico Monzino IRCCS in Milan (M) (Table S1). These variants were identified in 84 probands fulfilling the diagnostic criteria for HCM [1]. Genetic testing was performed using NGS-based gene panels, and suspicious variants were confirmed by Sanger sequencing. Variant classification was based on the standard ACMG/AMP guidelines [8] using data available at the time of the first clinical report (Round 1).

### 2.2. Reclassification Design

**Figure 1:**
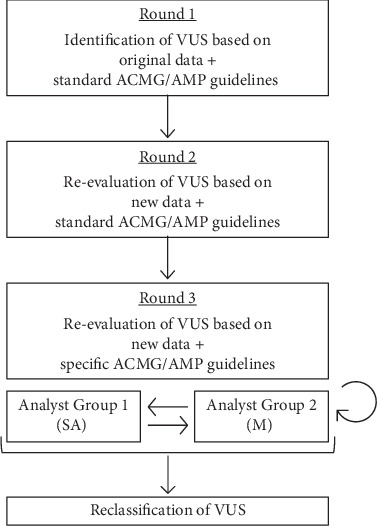
Workflow: Round 1 as the variant classification at the time of the first clinical report, Round 2 as the variant reclassification using the original ACMG/AMP framework with the recommendations by ClinGen's “Sequence Variant Interpretation Working Group” (SVI), and Round 3 as the variant reclassification using the ACMG/AMP specifications adapted for HCM. For the latter round, each variant was independently assessed by two groups of curators (SA/M) until a consensus was reached.

In order to determine whether new data or new specifications are more relevant to the reclassification over time [18, 19], the reassessment of VUS was conducted in two rounds (Figure 1), both based on the most recent information: “new data + standard ACMG/AMP guidelines” (Round 2) and “new data + specific ACMG/AMP guidelines” (Round 3), when it is possible to apply 20 out of 28 criteria, 13 toward pathogenicity and seven toward benignity, with fixed or variable strength. Moreover, each criterion is associated with a numerical score based on its strength of evidence for pathogenicity (positive values) or benignity (negative values), respectively (supporting = ±1, moderate = ±2, strong = ±4, and very strong/standalone = ±8) [20]. For each criterion, the sources used in this study are described in Table 1. The data collected are reported in Table S1.

To rule out operator bias and verify the standardization of interpretation, in Round 3, all reclassifications were performed by two groups of experienced variant curators (Figure 1). After the initial independent analysis, discordances were reviewed through consultations until a consensus was reached.

Once all the applicable criteria were concordantly assigned, the variants were classified into one of five classes (pathogenic, likely pathogenic, VUS, likely benign, or benign) [10] based on the combination rules or the sum of the associated scores (i.e., point approach), the latter especially when both benign and pathogenic criteria were applied conflictually (≤−7 = benign, −6 to − 2 = likely benign, −1 to 5 = VUS, 6 − 9 = likely pathogenic, and ≥10 = pathogenic).

In cases where VUSs were reclassified toward pathogenicity (P/LP) or benignity (B/LB), the time elapsed from initial report to reclassification was calculated, and both carrier patients and their families were critically re-evaluated.

To compare the classifications provided by this manual curation with others that are available, the main databases and tools for clinical interpretation of variants were reviewed. In particular, the eventual clinical significance reported in ClinVar (as a public archive of reports submitted by clinical and research laboratories, [21]) and CardioClassifier v0.2.0 (as an automated web tool for variant interpretation in inherited cardiac conditions, [22]) was checked and discussed (Table S1).

### 2.3. Cardio-Specifications Matcher (Cardio-SM)

To streamline and standardize the classification process, we developed a semiautomated decision support tool called *Cardio-SM*. The first step consisted of the parametrization of rule–gene pairs using a systematic tiered approach. Briefly, the criteria framework was grouped into nine main topics (population, prevalence, origin, type of variant, critical domain and/or mutational hotspot, functional data, bioinformatic prediction, segregation, and co-occurrence), and each topic responds to linked criteria that are activated based on the ACMG/AMP specifications adapted for HCM, as described in Table 1. Next, a user interface that includes visual elements, an organized layout, and user-friendly navigation pathways was built. By prompting the users to input relevant data and/or respond to targeted conceptual questions, this tool comprehensively guides them through the criteria assignment process. As output, *Cardio-SM* provides the variant classification based on the combination of applied criteria, considering both the standard and the point approach. Additionally, a summary with the reasons behind is generated in order to ensure downstream review and validation by the curator. This toolkit was developed in Python and is now available online at https://cardio-sm.org/.

## 3. Results

Between January 2017 and March 2024, a total of 558 HCM index cases from two independent cohorts underwent genetic testing. Among these, 84 cases (15.1%) harbored at least one variant classified as VUS in *MYBPC3*, *MYH7*, *TNNT2*, *TNNI3*, *TPM1*, *MYL2*, *MYL3*, and *ACTC1* genes (Round 1). The main features of VUS are reported in Table S1, based on the most recent data obtained.

By considering the standard ACMG/AMP + SVI framework, none of the VUSs were reclassified (Round 2). In particular, five out of 69 variants gained an advantage from new evidence published after their initial reporting. This resulted in the application of PP2 (i.e., etiological fraction data from [14]), PS3 (i.e., functional data from [23, 24]), and PM5 criteria (i.e., expert panel submission by [12]). In addition, four out of 69 variants have taken advantage of results based on segregation analysis to apply the PP1 code. However, all this new data was not sufficient to refine the interpretation and clarify the role of these variants (Table S1).

**Figure 2:**
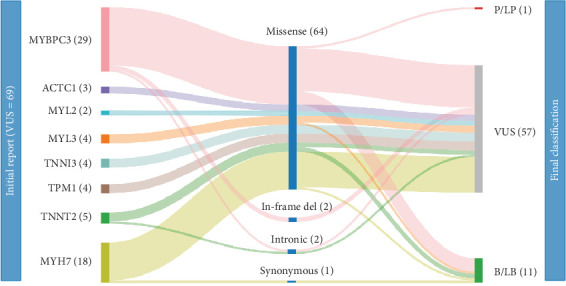
Sankey diagram depicting VUS according to genes, types of variants, and clinical significance. The initial report corresponds to the time of the first clinical report (Round 1), and the Final classification is based on the ACMG/AMP specifications adapted for HCM (Round 3). No reinterpretations were produced by the classification based on the original ACMG/AMP framework (Round 2). The number of variants for each level is given in brackets.

Using the ACMG/AMP specifications adapted for HCM, we reclassified 17.4% (*N* = 12/69, 95% CI: 10.2%–28.0%) of the original VUS (Round 3), which corresponds to a significant reduction in VUS burden from 69 to 57 (*p* < 0.01). Specifically, 91.7% (*N* = 11/12) were reclassified as B/LB and 8.3% (*N* = 1/12) as likely pathogenic (Figure 2). The genes that benefited the most were *TNNT2* (40%), *MYL3* (25%), *MYBPC3* (24%), and *MYH7* (11%) (Figure 2).

**Figure 3:**
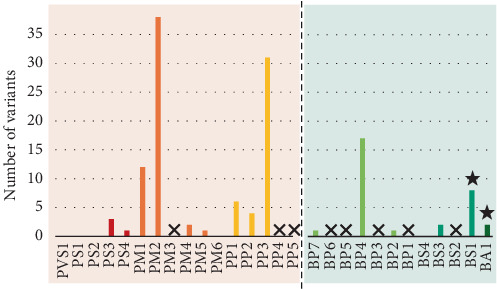
Criteria usage in the reassessment of variants. In detail, thirteen for pathogenicity (red nuances according to strength), seven for benignity (green nuances according to strength), and eight for nonapplicability (cross symbol). Star symbol = the most impactful criterion for reclassification.

The reclassifications have taken an average of 68.3 months (equal to 5.7 years) from initial reporting to date. The most frequently used criteria were as follows: population allele frequency (PM2 = 55%, BA1/BS1 = 14%), bioinformatic prediction (PP3 = 45%, BP4 = 25%), and variant location (PM1 = 21%). Of the 12 variants that were reclassified, the application of BA1/BS1 contributed to classification differences in over 90% of cases (*N* = 11/12). Other criteria were poorly used (PS3/BS3, PS4, PM5, PP1, and BP2) or never used (PS1, PS2/PM6, and BS4) (Figure 3). Furthermore, the point-based approach was adopted in 58.3% of variants to manage conflicting criteria, for which there is no standard combination rule. Reclassification results are detailed in Table 2.

From a methodological point of view, concordance between the two groups of curators was reached for 98.6% (*N* = 68/69) of variants and for 91.2% (*N* = 126/138) of criteria usage after the first consultation, reaching a consensus (100.0%, *N* = 69/69 variants and *N* = 131/131 criteria used) at the second consultation. In particular, the criteria discussed were PM2/BS1 (*N* = 7, ultimately removed) and PP1/BP2 (*N* = 5, ultimately added) (Table S1).

**Figure 4:**
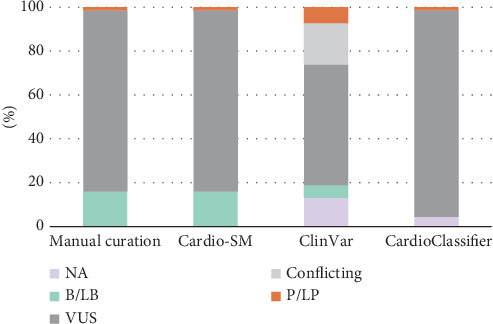
Distribution of variant classification, provided by manual curation, Cardio-SM tool, and public sources (ClinVar/CardioClassifier) between the five-tier system (reported as pathogenicity [P/LP], benignity [B/LB], uncertain [VUS] or conflicting classifications, or absent [NA]).

Furthermore, we tested the ability of Cardio-SM to support and standardize the correct use of the ACMG/AMP specifications for HCM. The manually curated variants were evaluated by a third independent curator. Using Cardio-SM, identical rule activations and variant classifications have been achieved (Figure 4).

Finally, an analysis was performed to compare our reclassification based on ACMG/AMP criteria specified for HCM with the main databases and tools as ClinVar and CardioClassifier (Figure 4). When comparing the assigned classifications with those reported in ClinVar (Table S1), the concordance rate was 81.2% (*N* = 56/69). The remaining 18.8% remains conflicting; in particular, eight variants were reclassified by our curation, downgrading them to benignity (13.3%), whereas five variants are classified as B/LB or P/LP in ClinVar.

Regarding CardioClassifier (Table S1), 11 out of 68 inconclusive variants were reclassified as B/LB (16.2%). Only one variant is already classified as likely pathogenic by CardioClassifier, which was also concordantly classified here. It is interesting to note the criteria applied, which were made explicit by CardioClassifier. Briefly, CardioClassifier applied 141 criteria, as reported in Table S1. Of those, 46.1% were also recognized in our reassessment (*N* = 65/141). However, the remaining criteria (53.9%, *N* = 76/141, mainly PM2 and PP2) were no longer applicable, due to more stringent thresholds in the ACMG/AMP specifications for HCM. Conversely, we applied 131 criteria, as reported in Table 2. Besides those shared (*N* = 65/131), the application of additional rules was allowed here (*N* = 66/131) by taking advantage of more accurate thresholds in the ACMG/AMP specifications for HCM.

## 4. Discussion

In this study, we present for the first time a real-world application of the ACMG/AMP criteria adapted for core sarcomeric genes to reinterpret VUS in the context of HCM. Here, we re-evaluated 69 VUSs in eight genes definitively associated with HCM (*MYBPC*3 = 29, *MYH*7 = 18, *TNNT*2 = 5, *TNNI*3 = 4, *TPM*1 = 4, *MYL*2 = 2, *MYL*3 = 4, and *ACTC*1 = 3; Figure 2) [5]. These variants were previously classified based on the standard ACMG/AMP guidelines [8] using data available at the time of the first clinical report (January 2017 to March 2024). This study resulted in a 17.4% reclassification rate involving 21.4% of HCM patients (*N* = 18/84), who can now benefit from dedicated clinical management. Therefore, these patients were recontacted and received counseling to clearly communicate the reinterpretation of their genetic results. In particular, the variants reclassified as B/LB were related to 17 probands, for which it was ensured that anxiety for an ambiguous result was reduced and trust in the medical system remained. For the proband carrying the variant reclassified as LP, clinical follow-up and family surveillance programs were explained. However, the rate of pathogenic conversion was low, highlighting how VUS may have a clinical impact on phenotype but lack evidence for being classified as P/LP variants [2].

Most VUSs were missense variants (92.8%), which are inherently more difficult to interpret [25]. Although bioinformatic prediction methods for evaluating the effects of missense variants have improved and have been applied for most variants (PP3/BP4 = 70%), this type of evidence as supporting strength is not sufficiently contributing to reclassification. Unfortunately, functional assays in sarcomeric genes (related to PS3/BS3) are rarely carried out and thus not often applied here (7%). Future development of more effective and high-throughput methods, such as those happening in genes encoding ion channels, may be required to improve variant interpretation [26].

Population evidence more frequently contributed to VUS reclassification, especially to benignity conversion due to standalone/strong strength. Although frequency data are usually available during initial classification, the fact that the Cardiomyopathy VCEP specifications provide more appropriate thresholds allowed for greater application of BA1/BS1 criteria.

Conversely, clinical evidence (spanning from prevalence to cosegregation) that would have an impactful role on directing classification was of limited use (PS4 = 1%, PP1/BS4 = 9%, and BP2 = 1%). Further efforts to generate new and more recent evidence, such as large case-control studies performed in hereditary cancer research [27], remain essential. Moreover, segregation data and co-occurrence evidence are often internal information of laboratories, and this was the reason behind the initial discordance between two groups of variant curators in this study. It may be useful to conduct segregation analysis on as many affected relatives as possible, by explaining that the scope is to further assess the clinical significance of a variant within individual families [28]. However, the genetic counseling should make it clear that the identification and segregation of a VUS in a family does not formally guide the medical management of either the proband or of family members. Pedigree information can also come from unpublished data or data published in an inaccurate manner and potentially causes disagreement among different providers and databases. Therefore, data sharing and peer-review initiatives should be encouraged.

Hence, this study shows the lack of evidence in the HCM context, including segregation data, functional assays, and case-control studies. This insufficiency of evidence prevents the criteria framework from being fully applied (PP1/BS4, PS3/BS3, and PS4). To improve this situation, solutions may be implemented. One solution remains investing to fill knowledge gaps. Another solution might be remodeling the criteria framework to better suit the needs of HCM. This could include expanding the five-tier classification system and/or introducing new degrees of strength for the criteria. In fact, although the ACMG/AMP system provides a robust standard for Mendelian variant interpretation, it may not fully capture the spectrum of genetic effects observed in complex disease architectures, where some alleles confer predisposition or risk and act in combination with other variants or modifiers [29, 30].

In recent years, the ACMG/AMP variant interpretation guidelines have already been updated by modifying the applicability and strength of several criteria. An interesting comparison can be made between the *MYH7* specifications from 2018 (Version 1.0.0) and those from 2024 (Version 2.0.0). While 19 criteria remain the same, nine criteria differ between the two versions. In detail, PVS1 and BP5 are no longer applicable, and PS3/BS3 can now be used with variable strength, as opposed to strong strength only, to incorporate as much evidence as possible. More appropriate thresholds are provided for PM1, BS1, and PP3/BP4. Notably, proband counting is no longer mentioned for applying PS4 in favor of the odds ratio (OR) (in particular, the lower bound of its 95% CI, lbOR), as obtained from case-control studies, which is inherently more accurate. However, this may be less effective. For example, the c.2572C > T variant in the *MYH7* gene could be classified as LP by adding PS4_Supporting (variant identified in 3 ≥ 2 probands [Version 1.0.0] vs. lbOR = 3.1 < 5 [Version 2.0.0]). Therefore, continuous updating and improvement of the gene-specific ACMG/AMP rules should be carried forward, particularly when classifying low-penetrance sarcomeric variants. This newly described type of sarcomeric variants [23, 31] is relatively common in the population. When isolated, they are associated with a mild HCM phenotype, while in combination with pathogenic sarcomeric variants, they increase the severity of the clinical phenotype, supporting an additive effect. A future perspective could be introducing a lower strength (e.g., PS4_Minimal Supporting with a point value of 0.5) that accurately encapsulates even the slightest prevalence effect (1 < lbOR < 5). Moreover, if BA1/BS1 is the only benign criterion present in addition to PS4_Minimal Supporting, its strength should be reduced proportionally. This would also enable the evaluation of variants recently described as “low-penetrance/intermediate effect” variants [23, 31] that have a high population frequency but are, however, associated with disease risk as *MYBPC3*:c.1321G > A, *MYL3*:c.170C > A, and *TNNT2*:c.862C > T, improving transparency for clinical reporting and comparability.

Promoting the standardization of variant classification also involves a more objective interpretability of the gene-specific ACMG/AMP rules. Even if these guidelines provide expert-based evidence, they introduce a layer of complexity with multiple data needed and leaving room for discretion on the part of the operator. To disseminate competence and ensure harmonization across curators, we developed the Cardio-SM. The tool has already set all the required rules and asks for targeted inputs each time. Moreover, it is designed to accommodate future extensions that more explicitly capture complex disease architectures. This makes the use of the criteria framework comprehensive, correct, and easy to update.

In conclusion, the reclassification using the Cardiomyopathy VCEP specifications leads to a significant reduction of the VUS number compared to reclassification using the standard ACMG/AMP classification system or most recent data alone. This supports the efforts for eliminating VUS in precision genomic medicine [32]. Nonetheless, the lack of key types of evidence and the ongoing need for interpretative guidance highlight the importance of consistently generating data, refining methods, and expertly curating information.

## Figures and Tables

**Table 1:** Proposed approach based on ACMG/AMP criteria specified for HCM, with the applicability rules defined by Cardiomyopathy VCEP and the sources used in this study. Not included are the criteria no longer applicable (PM3, PP4, PP5, BS2, BP1, BP3, BP5, and BP6).

**Type of evidence**	**Criteria**	**Strength (point)**	**Manner of assigning the rule**	**Applicability**	**Source**
Protein consequence (type of variants)	PVS1	Very strong (8)	Gene-specific	Null variant in a loss-of-function gene	Variant features
	PM4	Moderate (2)	Standard ACMG/AMP guidelines	In-frame del/ins or truncating variants when NA-PVS1	Variant features
	PP2	Supporting (1)	Gene-specific	Missense variant in a missense-intolerant gene	Variant features
	BP7	Supporting (−1)	Standard ACMG/AMP guidelines	Synonymous or deep intronic variant	Variant features

Protein consequence (mutational hotspot and/or critical domain)	PM1	Moderate (2)	Gene-specific	Missense variant included in the critical codons	Variant features
	PS1	Strong (4)	Standard ACMG/AMP guidelines	Other P/LP nucleotide change(s) for the same aminoacidic change	UCSC Genome Browser
	PM5	Moderate (2) and supporting (1)	Standard ACMG/AMP guidelines	Other P/LP aminoacidic change(s)	UCSC Genome Browser

Population	PM2	Supporting (1)	Disease-specific	Absent or upperAF ≤ 0.00004	gnomAD v.4.1
	BA1	Standalone (−8)	Disease-specific	FAF ≥ 0.001	gnomAD v.4.1
	BS1	Strong (−4)	Disease/gene-specific	FAF ≥ 0.0001 or FAF ≥ 0.0002	gnomAD v.4.1

Prevalence	PS4	Strong (4), moderate (2), and supporting (1)	Disease-specific	LowerOR ≥ 20, 10, 5	Walsh et al. [14]

Functional data	PS3	Strong (4), moderate (2), and supporting (1)	Disease-specific	Presence of RNA-based, in vivo, or in vitro studies	Literature
	BS3	Strong (−4), moderate (−2), and supporting (−1)	Disease-specific	Presence of RNA-based, in vivo, or in vitro studies	Literature

Bioinformatic prediction	PP3	Supporting (1)	Disease-specific	Revel ≥ 0.70 or SpliceAI ≥ 0.2	Ioannidis et al. [15] and Jaganathan et al. [16]
	BP4	Supporting (−1)	Disease-specific	Revel ≤ 0.40 and SpliceAI < 0.2	Ioannidis et al. [15] and Jaganathan et al. [16]

Origin	PS2	Strong (4)	Disease-specific	Proven de novo origin	Personal and family history
	PM6	Moderate (2)	Disease-specific	Presumed de novo origin	Personal and family history

Segregation	PP1	Strong (4), moderate (2), and supporting (1)	Disease-specific	≥ 7, 5, and 3 cosegregations with disease (meiosis counting)	Personal and family history + literature
	BS4	Strong (−4)	Disease-specific	≥ 2 nonsegregations with disease (meiosis counting)	Personal and family history + literature

Co-occurrence	BP2	Supporting (−1)	Disease-specific	Observed with other *p* variant(s) without a worse phenotype	Personal and family history

**Table 2:** List of VUS considered in this study and reclassified into one of five classes (pathogenic, likely pathogenic, VUS, likely benign, or benign) based on the combination rules or the sum of the associated scores (i.e., point-based approach).

**Gene (transcript)**	**HGVS Nucleotide**	**HGVS Protein**	**Applicable ACMG/AMP criteria specified for HCM (strength)**	**Total points**	**Final classification**	**Method used for classification**
NM_005159.5(ACTC1)	c.92C > T	p.(Ala31Val)	PP1_Supporting; PM2_Supporting; PP3	3	VUS	Combination rules
NM_005159.5(ACTC1)	c.855G > C	p.(Met285Ile)	PM2_Supporting; PP3	2	VUS	Combination rules
NM_005159.5(ACTC1)	c.886 T > C	p.(Tyr296His)	PM2_Supporting; PP3; PP1_Supporting	3	VUS	Combination rules
NM_000256.3(MYBPC3)	c.67G > A	p.(Ala23Thr)	BP4	−1	VUS	Combination rules
NM_000256.3(MYBPC3)	c.83 T > A	p.(Val28Glu)	PM2_Supporting; BP4	0	VUS	Combination rules
NM_000256.3(MYBPC3)	c.532G > A	p.(Val178Met)	PP3	1	VUS	Combination rules
**NM_000256.3(MYBPC3)**	**c.565G > A**	**p.(Val189Ile)**	**BA1; BP4**	−**9**	**Benign**	**Combination rules**
NM_000256.3(MYBPC3)	c.636C > G	p.(Ser212Arg)	PS3_Supporting; PM2_Supporting; PP3	3	VUS	Combination rules
NM_000256.3(MYBPC3)	c.659A > G	p.(Tyr220Cys)	BS3_Supporting	−1	VUS	Combination rules
**NM_000256.3(MYBPC3)**	**c.787G > A**	**p.(Gly263Arg)**	**BS1; BP4**	−**5**	**Likely benign**	**Combination rules**
NM_000256.3(MYBPC3)	c.1102_1104del	p.(Lys368del)	PM2_Supporting; PM4	3	VUS	Combination rules
NM_000256.3(MYBPC3)	c.1112C > G	p.(Pro371Arg)	PM2_Supporting; PP3	2	VUS	Combination rules
**NM_000256.3(MYBPC3)**	**c.1321G > A**	**p.(Glu441Lys)**	**BA1; BP4**	−**9**	**Benign**	**Combination rules**
**NM_000256.3(MYBPC3)**	**c.1471G > A**	**p.(Val491Met)**	**PM1; BS1**	−**2**	**Likely benign**	**Point-based approach**
**NM_000256.3(MYBPC3)**	**c.1484G > A**	**p.(Arg495Gln)**	**PS3_Moderate; PS4_Moderate; PM1**	**6**	**Likely pathogenic**	**Combination rules**
NM_000256.3(MYBPC3)	c.1591G > C	p.(Gly531Arg)	PS3_Supporting; PM2_Supporting; PP1_Supporting; PP3	4	VUS	Combination rules
NM_000256.3(MYBPC3)	c.1765C > T	p.(Arg589Cys)	PP3	1	VUS	Combination rules
NM_000256.3(MYBPC3)	c.2030C > T	p.(Pro677Leu)	PM2_Supporting; PP3	2	VUS	Combination rules
NM_000256.3(MYBPC3)	c.2117G > A	p.(Gly706Asp)	PM2_Supporting; BP4	0	VUS	Combination rules
NM_000256.3(MYBPC3)	c.2198G > T	p.(Arg733Leu)	PM2_Supporting; BP4	0	VUS	Combination rules
NM_000256.3(MYBPC3)	c.2210C > T	p.(Thr737Met)	BP4	−1	VUS	Combination rules
**NM_000256.3(MYBPC3)**	**c.2311G > A**	**p.(Val771Met)**	**BS1; BP4**	−**5**	**Likely benign**	**Combination rules**
NM_000256.3(MYBPC3)	c.2441_2443del	p.(Lys814del)	PM4; BS3_Supporting; BP4	0	VUS	Combination rules
NM_000256.3(MYBPC3)	c.2470G > A	p.(Asp824Asn)	BP4	−1	VUS	Combination rules
NM_000256.3(MYBPC3)	c.2716G > A	p.(Val906Met)	NA	0	VUS	Combination rules
NM_000256.3(MYBPC3)	c.3284C > T	p.(Thr1095Met)	BP4	−1	VUS	Combination rules
NM_000256.3(MYBPC3)	c.3364A > T	p.(Thr1122Ser)	BP2_Supporting	−1	VUS	Combination rules
NM_000256.3(MYBPC3)	c.3370 T > C	p.(Cys1124Arg)	PP3	1	VUS	Combination rules
**NM_000256.3(MYBPC3)**	**c.3413G > A**	**p.(Arg1138His)**	**PP3, BA1 ** ^**a**^	−**3**	**Likely benign**	**Point-based approach**
NM_000256.3(MYBPC3)	c.3614G > C	p.(Arg1205Pro)	PM2_Supporting; PP3	2	VUS	Combination rules
NM_000256.3(MYBPC3)	c.3758G > A	p.(Cys1253Tyr)	PP3; PM1; PM2_Supporting	4	VUS	Combination rules
NM_000256.3(MYBPC3)	c.3815-11_3815-9del	p.(?)	PM2_Supporting; PP3	2	VUS	Combination rules
NM_000257.4(MYH7)	c.138 T > A	p.(Phe46Leu)	PM2_Supporting	1	VUS	Combination rules
NM_000257.4(MYH7)	c.277C > G	p.(Leu93Val)	PM2_Supporting	1	VUS	Combination rules
NM_000257.4(MYH7)	c.328G > A	p.(Gly110Ser)	BP4	−1	VUS	Combination rules
NM_000257.4(MYH7)	c.697G > T	p.(Ala233Ser)	PM1; PM2_Supporting; PP3	4	VUS	Combination rules
NM_000257.4(MYH7)	c.1108G > A	p.(Glu370Lys)	PM1; PM2_Supporting; PP3	4	VUS	Combination rules
NM_000257.4(MYH7)	c.2548G > A	p.(Ala850Thr)	PM1; PM2_Supporting; PP3	4	VUS	Combination rules
NM_000257.4(MYH7)	c.2572C > T	p.(Arg858Cys)	PM1; PM2_Supporting; PP1_Moderate	5	VUS	Combination rules
NM_000257.4(MYH7)	c.3274G > A	p.(Ala1092Thr)	PM2_Supporting; BP4	0	VUS	Combination rules
NM_000257.4(MYH7)	c.3701A > C	p.(Asn1234Thr)	PM2_Supporting	1	VUS	Combination rules
NM_000257.4(MYH7)	c.4040A > G	p.(Tyr1347Cys)	NA	0	VUS	Combination rules
NM_000257.4(MYH7)	c.4159G > A	p.(Glu1387Lys)	PP3	1	VUS	Combination rules
NM_000257.4(MYH7)	c.4259G > A	p.(Arg1420Gln)	PM2_Supporting; PM5_Supporting; PP3	3	VUS	Combination rules
NM_000257.4(MYH7)	c.4985G > A	p.(Arg1662His)	NA	0	VUS	Combination rules
NM_000257.4(MYH7)	c.5066G > T	p.(Arg1689Leu)	PM2_Supporting; PP3	2	VUS	Combination rules
**NM_000257.4(MYH7)**	**c.5287G > A**	**p.(Ala1763Thr)**	**PP3; BS1**	−**3**	**Likely benign**	**Point-based approach**
NM_000257.4(MYH7)	c.5527A > G	p.(Ser1843Gly)	PM2_Supporting; BP4	0	VUS	Combination rules
**NM_000257.4(MYH7)**	**c.5562G > A**	**p.(Thr1854=)**	**PP3; BS1**	−**3**	**Likely benign**	**Point-based approach**
NM_000257.4(MYH7)	c.5724G > C	p.(Glu1908Asp)	PM2_Supporting	1	VUS	Combination rules
NM_000432.4(MYL2)	c.430C > G	p.(Pro144Ala)	PM2_Supporting	1	VUS	Combination rules
NM_000432.4(MYL2)	c.470A > G	p.(His157Arg)	PM2_Supporting	1	VUS	Combination rules
**NM_000258.3(MYL3)**	**c.170C > A**	**p.(Ala57Asp)**	**PP3; BS1**	−**3**	**Likely benign**	**Point-based approach**
NM_000258.3(MYL3)	c.187C > T	p.(Arg63Cys)	PP3	1	VUS	Combination rules
NM_000258.3(MYL3)	c.466G > A	p.(Val156Met)	PM2_Supporting; PP3	2	VUS	Combination rules
NM_000258.3(MYL3)	c.466G > T	p.(Val156Leu)	PM2_Supporting; PP3	2	VUS	Combination rules
NM_000363.5(TNNI3)	c.428C > A	p.(Thr143Asn)	PM1	2	VUS	Combination rules
NM_000363.5(TNNI3)	c.566G > A	p.(Gly189Glu)	PM2_Supporting; PM1; PP3	4	VUS	Combination rules
NM_000363.5(TNNI3)	c.581A > G	p.(Asn194Ser)	PM2_Supporting; PM1; PP3	4	VUS	Combination rules
NM_000363.5(TNNI3)	c.592C > G	p.(Leu198Val)	PM1; PM2_Supporting; BP4; PP1_Supporting	3	VUS	Combination rules
NM_001276345.2(TNNT2)	c.52+12C > T	p.(?)	PM2_Supporting; BP4; BP7	−1	VUS	Combination rules
**NM_001276345.2(TNNT2)**	**c.113C > T**	**p.(Ala38Val)**	**BS1**	−**4**	**Likely benign**	**Point-based approach**
NM_001276345.2(TNNT2)	c.341C > T	p.(Ala114Val)	PM1; PP3	3	VUS	Combination rules
NM_001276345.2(TNNT2)	c.815A > T	p.(Asn272Ile)	PM2_Supporting	1	VUS	Combination rules
**NM_001276345.2(TNNT2)**	**c.862C > T**	**p.(Arg288Cys)**	**PS3_Supporting; PP1_Supporting; BS1**	−**2**	**Likely benign**	**Point-based approach**
NM_001018005.2(TPM1)	c.24G > A	p.(Met8IIe)	PM2_Supporting; PP2	2	VUS	Combination rules
NM_001018005.2(TPM1)	c.82G > C	p.(Asp28His)	PP2	1	VUS	Combination rules
NM_001018005.2(TPM1)	c.513C > G	p.(Ile171Met)	PM2_Supporting; PP2; PP3	3	VUS	Combination rules
NM_001018005.2(TPM1)	c.652G > A	p.(Glu218Lys)	PM2_Supporting; PP2; PP3	3	VUS	Combination rules

*Note:* Bold font for variants reclassified toward benignity (B/LB) or pathogenicity (P/LP).

^a^This needed consideration related to a specific subpopulation.

## Data Availability

The data that support the findings of this study are available in the supporting information of this article and, where appropriate, from the corresponding author upon reasonable request.

## Supporting Information

Supporting Information Additional supporting information can be found online in the Supporting Information section. Table S1: The main features of VUS considered in this study, based on the most recent data collected.
